# Quantitative Analysis of Retinal Microvascular Perfusion and Novel Biomarkers of the Treatment Response in Diabetic Macular Edema

**DOI:** 10.1155/2020/2132037

**Published:** 2020-11-16

**Authors:** Young Gun Park, Young-Hoon Park

**Affiliations:** ^1^Department of Ophthalmology and Visual Science, Seoul St. Mary's Hospital, College of Medicine, The Catholic University of Korea, Seoul, Republic of Korea; ^2^Catholic Institute for Visual Science, College of Medicine, The Catholic University of Korea, Seoul, Republic of Korea

## Abstract

**Purpose:**

We aimed to assess the changes of retinal microvascular parameters using optical coherence tomography angiography (OCTA) between diabetes macular edema (DME) and controls. We assessed the changes between the baseline microvascular parameters and final treatment response in patients with DME, initially treated with intravitreal dexamethasone (DEX) implant followed by antivascular endothelial growth factor (VEGF) injections on an as-needed basis.

**Methods:**

This retrospective study included 90 DME patients and 24 healthy control subjects. All subjects had their best-corrected visual acuity (BCVA) and central macular thickness (CMT) measured at baseline and after 12 months. Vessel density (VD) in the superficial capillary plexus (SCP) and deep capillary plexus (DCP) and the deep/superficial flow ratio at baseline were analyzed. A subgroup analysis was used to compare the treatment response. A poor-response group was defined by five or more retreatments at 12 months.

**Results:**

BCVA and CMT showed a significant improvement at 12 months (all *p* < 0.001). The VD in the whole and parafoveal areas of the DCP was significantly reduced in DME patients compared to that in controls (all *p* < 0.05). The DCP/SCP flow ratio was also significantly reduced in the DME group (1.08 ± 0.03 vs. 1.05 ± 0.02, *p* = 0.001). In the subgroup analysis, the VD in the foveal and whole DCP areas was significantly lower in the poor-response group than that in the good-response group (*p* = 0.043 and *p* = 0.048, respectively). The DCP/SCP flow ratio was also significantly lower in the poor-response group (*p* = 0.011).

**Conclusion:**

DME correlated with significant retinal microvascular impairment in the DCP. A decreased DCP/SCP flow ratio was observed in patients with DME that exhibited a poor treatment response. Retinal microvascular parameters could predict the treatment response in DME and help optimize clinical outcomes.

## 1. Introduction

Diabetic macular edema (DME), macular thickening due to diabetic retinopathy (DR), can present at any stage of this disease. It is caused by a blood-retinal barrier defect that leads to vascular leakage and fluid accumulation [1]. This process is the outcome of the expression of inflammatory factors, including vascular endothelial growth factor (VEGF), intercellular adhesion molecule-1, interleukin-6, and monocyte chemotactic protein-1, and leukostasis [2, 3]. Because DME can cause vision loss in severe cases, it is becoming an important public health issue [4].

Many different treatment options for DME have been developed, including anti-VEGF agents and corticosteroids [5, 6]. Intravitreal injection of anti-VEGF agents is a standard treatment for DME approved by the United States Food and Drug Administration [5]. However, this treatment poses a heavy financial burden on patients because of the numerous required injections during the year. On the other hand, intravitreal dexamethasone (DEX) implants (0.7 mg) (Ozurdex, Allergan, Inc., Irvine, CA, USA) consist of a biodegradable copolymer that slowly releases steroids over a period of approximately 4–6 months [7].

Recently, with the increased use of optical coherence tomography (OCT) and OCT angiography (OCTA), several studies have reported various imaging biomarkers and their association with the treatment response in DME [8, 9]. OCTA allows the acquisition of images of the retinal microvasculature with good reproducibility and repeatability in a safe, rapid, and noninvasive manner. However, OCTA studies on the foveal microvascular impairment in DME are limited compared with those using OCT. Previous OCTA studies have only reported on the changes in the foveal avascular zone and the impairment of foveal microcirculation in eyes with DR [10, 11].

Based on these results, we aimed to evaluate the changes in retinal microvascular parameters between DME patients and healthy controls. We assessed the differences between the baseline microvascular parameters and final treatment response in DME patients, initially treated with intravitreal DEX implant followed by anti-VEGF injections on an as-needed basis.

## 2. Materials and Methods

### 2.1. Ethical Considerations

All procedures were conducted in accordance with the Declaration of Helsinki and its later amendments. The study was approved by the Ethics Committee of Seoul St. Mary's Hospital and the Catholic University of Korea. The requirement for informed patient consent was waived due to the retrospective design of the study.

### 2.2. Study Design and Subjects

This study was a retrospective review of consecutive patients who attended the Department of Ophthalmology of Seoul St. Mary's Hospital between January 2017 and January 2019. The study included patients with a diagnosis of diabetes mellitus and DME, who received a DEX implant injection and were followed up for at least 12 months. The healthy control group included healthy patients with no posterior segment abnormalities or systemic comorbidities who attended medical checkups.

The exclusion criteria for patients with DME were as follows: (1) any other ocular disease that may affect ocular circulation (e.g., glaucoma, age-related macular degeneration, and refractive error > 5 diopters); (2) intraocular pressure > 25 mmHg; (3) severe media opacity (e.g., lens opacity due to cataract or thick asteroid hyalosis); (4) macular edema due to any other condition (such as retinal vessel obstruction or macular ischemia); and (5) history of retinal treatment, including laser, intravitreal injections, and other subtenon steroid injections, within 6 months before baseline evaluation.

All patients and controls had their best-corrected visual acuity (BCVA) measured initially and then underwent standardized dilated fundus examinations, including measurements by swept-source OCT and OCTA imaging (DRI OCT Triton; Topcon, Tokyo, Japan). In all patients, reevaluation after treatment was scheduled (as per usual clinical practice) 2 months after DEX implantation or 1 month after intravitreal anti-VEGF agent injection.

### 2.3. Treatment Protocol

The initial DEX implant (0.7 mg) was injected into the vitreous cavity using standard protocols. After the first DEX implant injection, patients received a second treatment with anti-VEGF agents (Avastin®; Genentech, Inc., San Francisco, CA) on an as-needed basis—pro re nata (PRN) dosing the regimen. The retreatment criterion was defined as central macular thickness(CMT) > 300 *μ*m.

Patients were classified into two groups according to their treatment response: good-response group, comprising patients who received 4 or fewer treatments, and poor-response group, comprising patients who received more than 5 treatments over 12 months from the initial treatment.

### 2.4. OCT Measurements

Swept-source OCT (DRI OCT Triton; Topcon) is a high-quality fundus imaging technique that relies on active eye tracking. The OCT images were generated using the horizontal OCT cross section (25 lines spaced 240 mm apart). CMT was measured using swept-source OCT (DRI OCT, Topcon, Japan). DME was defined as a CMT > 300 *μ*m.

### 2.5. OCTA Imaging and Analysis

OCTA was performed using the same device (DRI OCT Triton; Topcon). This device has an A-scan rate of 70,000 scans/s with an 840 nm wavelength light source and a 45 nm bandwidth. Patients with low-quality images (signal strength index < 50) were excluded. OCTA images of the superficial capillary plexus (SCP), deep capillary plexus (DCP), and the choriocapillaris network were generated and segmented automatically by the built-in software (IMAGEnet 6, version 1.25). SCP was delineated by 2.6 *μ*m below the internal limiting membrane to 15.6 *μ*m below the junction, between the inner plexiform and the inner nuclear layers; DCP was delineated by 15.6 *μ*m below the inner plexiform and the inner nuclear layers to 70.2 *μ*m below them. Large intraretinal cysts in DME are often involved in multilayers and lead to inaccurate segmentation errors. For eyes with incorrect segmentation, we manually adjusted the offset value of the inner and outer borders for the DCP so that the DCP slab was segmented from just below the inner plexiform layer to just below the outer plexiform layer. Vessel density was defined as the percentage area occupied by vessels in a circular region centered on the center of the foveal avascular zone. Whole en face, foveal, and parafoveal vessel density of the SCP and DCP within 1 and 3 mm inner and outer circles was measured using computer software. We calculated the flow ratio as the ratio of the vessel density in the DCP to that in the SCP (flow ratio = DCP vessel density/SCP vessel density) and determined the average values [12].

### 2.6. Statistical Analysis

Shapiro-Wilk test was used to assess the normality of data. A repeated measures ANOVA was used for the determination of the changes in visual acuity and in foveal thickness into the study patients. Unpaired *t*-tests were used for between-group comparisons. Correlations between OCT parameters and the number of retreatments were assessed using Pearson's correlation test. Univariate and multivariate logistic regression models were used to identify the potential factors associated with retreatment. The receiver operating characteristic (ROC) curve and the area under the curve were used to assess the predictability of the DCP/SCP ratio for good treatment response in DME. *p* values < 0.05 were considered statistically significant. All analyses were performed using commercial software (SPSS version 22.0; IBM, Armonk, NY, USA).

## 3. Results

### 3.1. Patients' Characteristics

In total, 90 eyes of 90 patients (38 women and 52 men) with DME treated with DEX implant injection and 25 eyes of 25 age-matched control subjects were included. The baseline clinical and demographic characteristics of all study subjects are shown in Table 1.

### 3.2. Clinical and OCT Parameters

**Figure 1:**
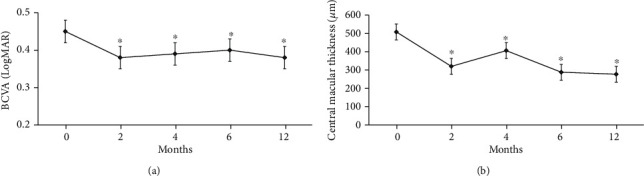
(a, b) Best-corrected visual acuity (BCVA) and central macular thickness (CMT) improved significantly compared to baseline during the observation period. ^∗^*p* < 0.001; repeated measures ANOVA.

The mean BCVA significantly improved from 0.45 ± 0.22 LogMAR at baseline to 0.38 ± 0.15 LogMAR after 12 months of treatment (*p* < 0.001). Moreover, the mean CMT significantly reduced from 508.07 ± 99.78 *μ*m at baseline to 277.04 ± 65.23 *μ*m (*p* < 0.001) after 12 months of treatment. (Figure 1) The average time from intravitreal DEX injection to anti-VEGF was 4.33 ± 1.13 months (range 3-8). The total number of injections was 4.79 ± 1.35.

### 3.3. OCTA Parameters

**Figure 2:**
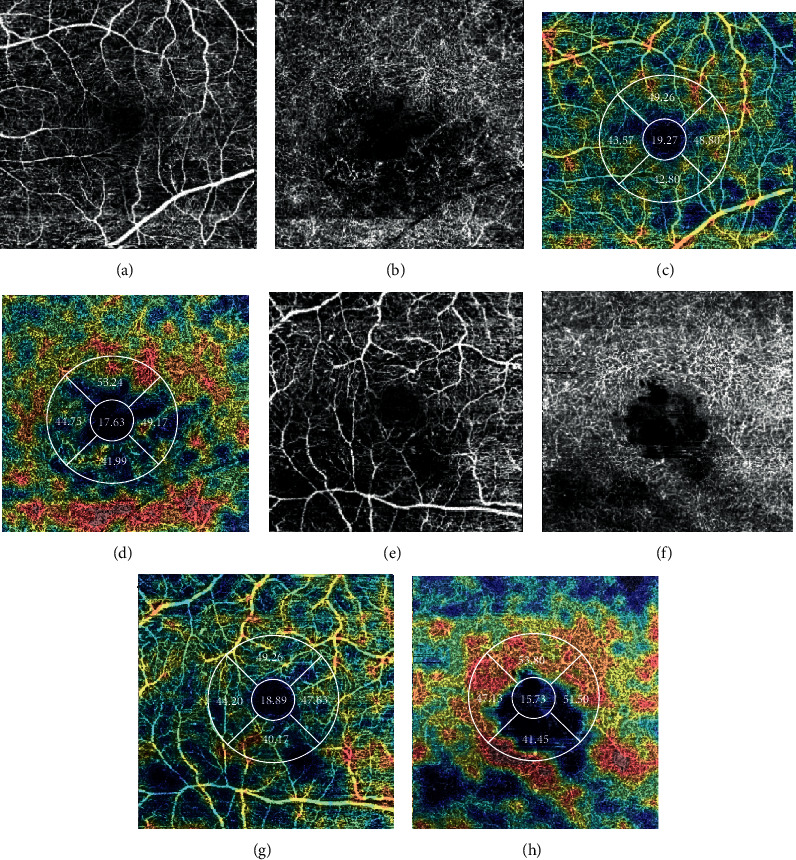
Representative samples of the microvascular parameters relative to the treatment response: (a–d) the parafoveal vessel density in the SCP and DCP (good-response group); (e–h) the parafoveal vessel density in the SCP and DCP (poor-response group).

Table 2 shows the SCP and DCP vessel density—whole, foveal, and parafoveal (superior/inferior/nasal/temporal)—in all patients. A statistically significant difference was observed between the DME and control groups in the microvascular parameters (Figure 2). In particular, the retinal vessel density in the whole and parafoveal areas of the DCP was significantly reduced in patients with DME compared with that in controls (all *p* < 0.05). In the SCP, only the whole retinal vessel density was significantly reduced in patients with DME (*p* = 0.026). The DCP/SCP flow ratio was also significantly reduced in the DME group (1.08 ± 0.03 vs. 1.05 ± 0.02, *p* = 0.001).

### 3.4. Correlation Analysis

Among the OCTA parameters, the whole area vessel density in the DCP and the DCP/SCP flow ratio correlated significantly with the number of injections received (*p* = 0.015, *r* = −0.415 and *p* = 0.025, *r* = −0.336, respectively).

In the multivariate linear regression analyses for identifying factors related to the number of injections received, only the glycated hemoglobin level among the clinical parameters and the DCP/SCP flow ratio among the OCTA parameters showed a significant association (*β* = −0.36, *p* = 0.047 and *β* = −1.003, *p* = 0.017, respectively).

### 3.5. Subgroup Analysis

**Figure 3:**
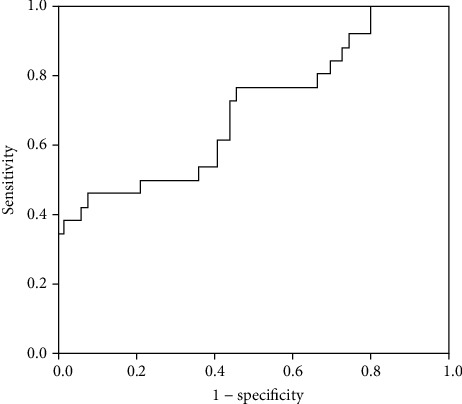
The ROC curve of the DCP/SCP flow ratio for predicting the treatment response. The area under the ROC curve was 0.703. ROC: receiver operating characteristics; DCP: deep capillary plexus; SCP: superficial capillary plexus.

There were 26 eyes in the good-response group and 64 eyes in the poor-response group. The foveal and whole vessel density in the DCP was significantly lower in the poor-response group than in the good-response group (*p* = 0.043 and *p* = 0.048, respectively) (Figure 1). Moreover, the DCP/SCP flow ratio was significantly lower in the poor-response group (*p* = 0.011) (Table 3). The ROC curve of the DCP/SCP flow ratio as a biomarker to predict poor treatment response is shown in Figure 3. The area under the curve was 0.702. No significant differences were observed between the two groups in the SCP vessel density and the DCP parafoveal vessel density.

### 3.6. Side Effects

No injection-related complications were observed. Four eyes developed ocular hypertension and were treated with antihypertensive drops. Two of the phakic eyes (6%) underwent cataract surgery due to the progression of lens opacity.

## 4. Discussion

In this study, we evaluated the OCTA microvascular parameters after a single intravitreal DEX implant injection followed by anti-VEGF therapy on a PRN basis and investigated their correlation with the number of injections received. DME was associated with significant retinal microvascular impairment in the DCP. There was a significant BCVA improvement and CMT reduction 12 months after the initial treatment. In patients with DME who had poor treatment response, there was a decreased DCP/SCP flow ratio.

We demonstrated that intravitreal DEX implant injection as initial therapy combined with anti-VEGF therapy on a PRN basis is effective for treating DME. In our study, we classified patients in the two response groups based on the number of treatments received. Busch et al. reported the outcomes of continued anti-VEGF therapy compared to switching DEX implant in eyes with DME in a real-world setting. At 12 months, mean anti-VEGF injections were 4.2 ± 2.4 except for the 3 loading injections. Therefore, we selected five treatments as the cutoff value [13, 14].

The pathophysiology of DME is complex and multifactorial; currently considered to be a chronic, low-grade inflammatory disorder [15]. It is associated with various vascular, neural, and glial cell components in the retina. Chronic hyperglycemia induces activation of the retinal glial cells that secrete VEGF and proinflammatory cytokines, leading to disruption in the blood-retinal barrier. Two widely used therapeutic strategies are the intravitreal steroid and intravitreal anti-VEGF injections [16–18].

Anti-VEGF agents, including ranibizumab, aflibercept, and bevacizumab, have been widely used for the treatment of DME for decades because of their convenience. However, the frequent injections due to the short duration of effect and the associated costs may be a heavy burden on the patients. In real-life settings, the treat-and-extend or PRN regimens have become popular as they decrease the treatment burden [18, 19].

DEX implants have demonstrated efficacy in the treatment of persistent DME, resistant to anti-VEGF treatment. Additionally, their effect is longer than that of anti-VEGF agents and six times stronger than that of triamcinolone acetonide [20, 21]. Several studies have reported on the use of intravitreal DEX as initial therapy in patients with DME [22–24].

In the present study, we compared the degree of microvascular damage in relation to the treatment response and the DCP/SCP flow ratio was found to represent a response index. The vessel density in the whole area in the DCP and the DCP/SCP flow ratio correlated significantly with the number of injections. Furthermore, the DCP/SCP flow ratio was the only OCTA parameter significantly related to the number of injections in the multivariate linear regression analyses. Considering that the vessel density in OCTA demonstrates personal variations, the DCP/SCP flow ratio is remarkable. Yeung et al. reported that the DCP/SCP flow ratio in patients with branch retinal vein occlusion was associated with the treatment response (*p* = 0.015) and suggested that this ratio can represent the relative damage of the DCP to that of the SCP in BRVO [12]. We wondered that the hypothesis that the capillary loss in the DCP is more prominent than that in the SCP in DR may be relatively acceptable. Instead of absolute values in the individual layer of the capillary plexus, the ratio of the vessel density in the DCP to that in the SCP may be more meaningful.

In a previous study, Moon et al. reported that the DCP loss was more prominent in DME eyes than in non-DME eyes [25, 26]. This trend was observed in anti-VEGF nonresponders compared with anti-VEGF responders. Altered vessel density in the DCP may imply a preference for this part of the retinal vascular system in the pathogenesis of DR or DME, as retinal venules originate from the deep retinal vascular layers. The earlier alterations in the deep vascular layer probably demonstrate retinal venular widening, damage to the capillary endings, and microaneurysms. It may also influence the breakdown of the blood-retinal barrier and the presence of DME [27–29].

In addition, we compared the two groups based on the number of treatment injections. The foveal and whole vessel density in the DCP and the DCP/SCP flow ratio were significantly lower in the poor-response group than in the good-response group. The underlying mechanism of the association between the low vessel density in the DCP and treatment inefficacy remains to be clearly defined. One possibility is excessive fluid flux from the vessels to the tissue because of the breakdown of the blood-retinal barrier caused by damage to the capillary endothelial tight junctions or various inflammatory cytokines [30, 31]. The other suggestion is that the DCP might play a part in the removal of excess fluid from the retina. The smaller vessel density in the DCP could lead to fluid accumulation in the retina and would reduce fluid absorption [32, 33].

To reduce ineffective repeated injections, it is important to screen for treatment-resistant DME in baseline examinations [14, 34]. However, it is difficult to directly compare the result of various treatment regimens. Ebneter et al. reported a comparison between a PRN and a treat-and-extend regimen in managing DME with intravitreal ranibizumab [35]. The mean number of injections was significantly different between the groups (PRN group: 5.9 ± 1.8; treat-and-extend group: 8.9 ± 2.0; *p* < 0.001). The VA of both groups showed similar gains (8.3 ± 6.7 vs. 9.3 ± 8.9, *p* = 0.3). In considering the duration of DEX implants, the number of retreatments in our study is not inferior to these results (3.46 ± 0.49 vs. 5.47 ± 0.9).

The most remarkable finding in our study was that the number of retreatments was related to the DCP/SCP flow ratio. Although several studies have evaluated the hard exudate or the microstructure with OCT to identify variables predicting the treatment response, we focused on the retinal microvascular changes evaluated with OCTA at baseline. In general, retinal vessel density was significantly reduced in patients with DME compared with that in controls. Compared with the SCP, vessel density rarefaction was prominent in the DCP, with the telangiectatic appearance of the retinal vessels. If the damage in the DCP is more severe than that in the SCP (in other words, considering the DCP/SCP ratio), the treatment response may be poor. Further studies are needed to identify predictors of the response to DME therapies and determine individualized therapeutic strategies according to the patients' features.

Our study has several limitations. First, the limited number of evaluated eyes led to low statistical power. Second, a selection bias may exist due to the retrospective study design. Third, most patients had a previous history of treatment and it may affect the outcomes. Fourth, the OCTA image artifacts can interfere with an accurate assessment of the actual status of the retinal microvasculatures. Projection artifacts might affect the visualization of the deep layer, and bias of segmentation error in cystoid macular edema may occur despite our best efforts to minimize such an effect. Last, it will be useful to observe the OCTA parameter changes, before and after the 12-month treatment. However, it is difficult to consistently examine OCTA during follow-up periods in real-life practice. Regardless, this report provides a framework for further research.

## 5. Conclusions

The results of our study suggested that the OCTA retinal microvascular parameters at baseline influence the treatment outcomes in DME. Furthermore, decreased DCP/SCP flow ratio was observed in patients with DME who manifested poor treatment response. These parameters could represent predictors of the treatment response and would help to optimize the clinical outcomes.

## Figures and Tables

**Table 1:** Baseline characteristics of the diabetic macular edema study population.

Characteristics	
Age	59.85 ± 9.04
Sex (M : F)	52 : 38
DM duration (yr)	13.46 ± 7.32
HbA1c	7.14 ± 0.66
DR grade	
Mild	0
Moderate	3
Severe NPDR	48
PDR	39
Hx of photocoagulation	62
No. of treatment-naïve patients	8 (8.9%)
BCVA (LogMAR)	0.45 ± 0.22
Phakic eyes	33 (37%)
Central macular thickness (*μ*m)	508.07 ± 99.78

Data are presented as means ± standarddeviation, numbers, or numbers (percentages). DM: diabetes mellitus; HbA1c: glycated hemoglobin; DR: diabetic retinopathy; NPDR: nonproliferative diabetic retinopathy; PDR: proliferative diabetic retinopathy; Hx: history; BCVA: best-corrected visual acuity; LogMAR: logarithm of the minimum angle of resolution.

**Table 2:** Vessel density in patients with DME and controls.

Characteristics	DME	Control	*p* value
*Superficial capillary plexus*			
Whole vessel density	44.58 ± 3.19	46.04 ± 2.29	0.026^∗^
Foveal vessel density	15.34 ± 5.45	15.23 ± 3.31	0.583
Parafoveal vessel density			
Superior	46.65 ± 4.95	48.7 ± 4.54	0.067
Inferior	45.72 ± 5.27	47.40 ± 5.73	0.167
Temporal	44.01 ± 4.60	45.74 ± 3.31	0.102
Nasal	41.94 ± 5.27	43.32 ± 3.94	0.243
*Deep capillary plexus*			
Whole vessel density	46.21 ± 3.01	49.55 ± 3.01	0.001^∗^
Foveal vessel density	14.46 ± 5.66	14.50 ± 2.74	0.389
Parafoveal vessel density			
Superior	48.26 ± 5.10	52.51 ± 4.64	0.001^∗^
Inferior	47.33 ± 4.94	50.86 ± 5.78	0.01^∗^
Temporal	45.42 ± 4.17	48.46 ± 3.81	0.002^∗^
Nasal	43.83 ± 4.94	46.38 ± 4.15	0.016^∗^
DCP/SCP ratio	1.05 ± 0.02	1.08 ± 0.03	0.001^∗^

Data are presented as means ± standarddeviation. DME: diabetic macular edema; DCP: deep capillary plexus; SCP: superficial capillary plexus.

**Table 3:** Clinical characteristics according to the DME treatment response.

Characteristics	Good-response group	Poor-response group	*p* value
Superficial capillary plexus			
Whole vessel density	44.80 ± 2.71	44.49 ± 3.34	0.41
Foveal vessel density	13.76 ± 4.81	15.98 ± 5.52	0.063
Parafoveal vessel density			
Superior	47.74 ± 5.23	46.2 ± 4.73	0.219
Inferior	45.99 ± 4.62	45.61 ± 5.47	0.725
Temporal	42.91 ± 4.42	44.46 ± 4.56	0.192
Nasal	42.58 ± 3.23	41.67 ± 5.13	0.51
Deep capillary plexus			
Whole vessel density	47.13 ± 2.12	45.83 ± 3.20	0.043^∗^
Foveal vessel density	13.08 ± 5.39	15.03 ± 5.62	0.048^∗^
Parafoveal vessel density			
Superior	49.5 ± 4.95	47.66 ± 5.01	0.094
Inferior	48.44 ± 4.41	46.87 ± 5.72	0.31
Temporal	44.89 ± 3.85	45.64 ± 4.24	0.504
Nasal	45.45 ± 3.35	43.17 ± 5.28	0.081
DCP/SCP ratio	1.05 ± 0.02	1.02 ± 0.03	0.011^∗^
Number of injections	3.46 ± 0.49	5.47 ± 0.9	0.03^∗^

Data are presented as means ± standarddeviation. DME: diabetic macular edema; DCP: deep capillary plexus; SCP: superficial capillary plexus.

## Data Availability

The data used to support the findings of this study are available from the corresponding author upon request.
